# Evolutionary and Biological Implications of Dental Mesial Drift in Rodents: The Case of the Ctenodactylidae (Rodentia, Mammalia)

**DOI:** 10.1371/journal.pone.0050197

**Published:** 2012-11-21

**Authors:** Helder Gomes Rodrigues, Floréal Solé, Cyril Charles, Paul Tafforeau, Monique Vianey-Liaud, Laurent Viriot

**Affiliations:** 1 Team “Evo-Devo of Vertebrate Dentition”, Institut de Génomique Fonctionnelle de Lyon, Unité Mixte de Recherche 5242 Centre National de la Recherche Scientifique, Ecole Normale Supérieure de Lyon, Université Claude Bernard Lyon 1, Lyon, France; 2 European Synchrotron Radiation Facility, Grenoble, France; 3 Laboratoire de Paléontologie, Institut des Sciences de l’Évolution de Montpellier, Unité Mixte de Recherche 5554 Centre National de la Recherche Scientifique, Université Montpellier 2, Montpellier, France; Monash University, Australia

## Abstract

Dental characters are importantly used for reconstructing the evolutionary history of mammals, because teeth represent the most abundant material available for the fossil species. However, the characteristics of dental renewal are presently poorly used, probably because dental formulae are frequently not properly established, whereas they could be of high interest for evolutionary and developmental issues. One of the oldest rodent families, the Ctenodactylidae, is intriguing in having longstanding disputed dental formulae. Here, we investigated 70 skulls among all extant ctenodactylid genera (*Ctenodactylus*, *Felovia*, *Massoutiera* and *Pectinator*) by using X-ray conventional and synchrotron microtomography in order to solve and discuss these dental issues. Our study clearly indicates that *Massoutiera*, *Felovia* and *Ctenodactylus* differ from *Pectinator* not only by a more derived dentition, but also by a more derived eruptive sequence. In addition to molars, their dentition only includes the fourth deciduous premolars, and no longer bears permanent premolars, conversely to *Pectinator*. Moreover, we found that these premolars are lost during adulthood, because of mesial drift of molars. Mesial drift is a striking mechanism involving migration of teeth allowed by both bone remodeling and dental resorption. This dental innovation is to date poorly known in rodents, since it is only the second report described. Interestingly, we noted that dental drift in rodents is always associated with high-crowned teeth favoring molar size enlargement. It can thus represent another adaptation to withstand high wear, inasmuch as these rodents inhabit desert environments where dust is abundant. A more accurate study of mesial drift in rodents would be very promising from evolutionary, biological and orthodontic points of view.

## Introduction

The interest of studying rodents among all mammals is stressed by their ecological ubiquity, coupled to their flourishing diversity (about 2300 species [1]). The evolutionary success of rodents is probably due to their small size, their high reproductive rates, their short breeding cycle, and their extensive range of dental characteristics. These dental particularities notably rely on the very high number of crown morphologies [2], [3], [4], enamel microstructure patterns [5], [6], and masticatory functions [7], [8], [9]. These variations have been extensively described in both extant and extinct forms. However, the mechanisms involved in the formation and maintenance of the dentition (i.e. development, eruption, replacement) remain to be accurately documented in rodents. Studies concerning this topic have mainly dealt with the mouse [10], [11], [12], [13], the usual model for mammalian biology. To date, we lack a global view regarding the diversity of mechanisms associated with rodent dentitions, and only rare discoveries showed very innovative dental systems in rodents [14]. In this context, this study aims at better understanding the underlying mechanisms of the establishment and replacement of the dentition of gundis (Ctenodactylidae), whose extant species present peculiar dental formulae. Their study might permit the opening of a new window on the knowledge of these dental issues.

The Ctenodactylidae encompass four endemic African genera: *Ctenodactylus*, *Felovia*, *Massoutiera*, and *Pectinator*. Originally, it was a highly diversified Asian group, notably during the Oligocene period (33.9–23 Ma; [15], [16], [17]). Then, this group dispersed into Europe, Arabia and Africa during the Miocene (23–5.3 Ma). Their evolutionary framework has been recently discussed in two main phylogenetical studies, involving all the ctenodactylid genera on one hand [18], and the crown group Ctenodactylinae on the other hand [19]. The dentition of extant ctenodactylids is characterized by high-crowned teeth covered by an important layer of cementum. They generally present one or two premolars and three molars in each jaw quadrant. One of the most striking issues is the potential presence of a third lower premolar (P_3_ or dP_3_, if deciduous) in *Pectinator* [18], [20], [21]. Indeed, a P_3_ was never observed in Rodentia, while this tooth is present in their ancestors and in their closest relatives, the Lagomorpha (i.e. rabbits and hares). However, detailed studies of early dental development in mice and squirrels demonstrated the occurrence of rudimentary dental buds developing in the diastemal area of the mandible, in front of presumptive functional teeth [22], [23], [24]. As the mineralization of these rudimentary buds is disrupted, they were assigned to aborted germs of premolars lost over evolution [25], [26]. Inasmuch as the complete development of a P_3_ might be still possible (e.g. beginning of mineralization in squirrels, which have one of the most primitive dentitions among rodents), the confirmation of the occurrence of a P_3_ in *Pectinator* could be of high interest for a better understanding of the underlying developmental processes involved in the reduction of mammalian dentitions.

Questions also arise concerning the actual occurrence of permanent premolars replacing the fourth upper and lower deciduous premolars (dP^4^ and dP_4_), and concerning their loss. In fact, only molars are present in adult specimens of *Ctenodactylus*, *Felovia* and *Massoutiera* [2], [18], [21], [27], [28]. The loss of premolars during the beginning of the adulthood is not rare in mammals. It was mentioned for instance in elephants, sirenians, kangaroos and wallabies [29], [30], [31], [32], [33]. These losses are induced by the forward pushing action of the erupting molars at the rear of the jaw, which leads to the mesial drift of all the cheek teeth, coupled to the remodeling of the surrounding alveolar bone. Then, the most anterior teeth, which are premolars, no longer fit within the jaw and are pushed out of the dental row. Since mesial drift has been recently found in rodents, and more precisely in African mole-rats (Bathyergidae [14]), we can hypothesize that a comparable mechanism is involved in the early loss of premolars in Ctenodactylidae.

The aim of this study is to more precisely describe the unusual dental characteristics of each extant ctenodactylid, and try to identify the mechanisms involved (e.g. mesial drift). These analyses notably benefit from high resolution microtomographic data of the dentitions at various ages which allow to precisely investigate the dental development, replacement, loss and possible drift.

## Materials and Methods

In this study, 22 skulls of *Ctenodactylus gundi*, 10 skulls of *Felovia vae*, 21 skulls of *Massoutiera mzabi* and 17 skulls of *Pectinator spekei* were investigated. These investigated specimens are housed in the Museum National d’Histoire Naturelle (MNHN) of Paris (France), and in the Naturhistorisches Museum of Basel (Switzerland).

High quality images of one or two skulls of each species were obtained using propagation phase contrast X-ray synchrotron microtomography at the European Synchrotron Radiation Facility (ESRF, Grenoble, France). Experiments were performed on the beamline BM5. One skull (*C. gundi* MNHN CG 1986-255) was scanned in 2008 using a monochromatic beam set at 25 keV using a double crystal Si111 Bragg monochromator. We used an indirect detector based on a 10 µm thick gadolinium oxide scintillator coupled with lenses based optic to a FReLoN CCD camera (Fast Readout Low Noise Charge Coupled Device). This system provides an isotropic voxel size of 7.39 µm. In order to have moderate phase contrast effect, we used a propagation distance of 500 mm. These data were reconstructed using filtered-backprojection algorithm, without phase retrieval, hence in edge detection mode. All the other specimens scanned at the ESRF for this study were imaged also on BM5 beamline in 2011, by using a pink beam configuration obtained by combination of a 125 µm thick LuAG scintillator and a lead glass based filter (equivalent to 0.7 mm of lead). Low energies of the spectrum were removed with 3 mm of aluminum and 2 mm of copper. Thanks to respective Kedge of the scintillator (63.31 keV) and filter (88 keV) and to the BM5 spectral properties, this configuration delivers a beam in which most of the detected photons are in the energy range between these two Kedges. It provides a quite narrow bandwidth (pink beam), allowing rapid high quality scans in propagation phase contrast mode (900 mm of propagation), without any effect of beam hardening due to the low absorption by the sample. The relatively high energy used for these scans is not problematic as the use of phase contrast brings a very high level of information. In order to make segmentation of the data more efficient, a single distance phase retrieval process was used [34]–[35] to reconstruct data linked to mineral density without the edge detection effect. Synchrotron microtomography has been proven to be very useful for very precise imaging of small elements, such as teeth [14]. The use of high quality pink beam coupled with single distance phase retrieval allows, for this type of sample, quality of data comparable to monochromatic beam, but with acquisition times 5 to 10 times shorter due to the higher flux of photons. One skull (*P. spekei*, MNHN CG 1995-19) was imaged using a GE phoenix nanotom 180 at energy of 100 keV with a cubic voxel of 5.64 µm. 3D renderings and virtual slices were then performed using VGStudio Max 2.0 software. Non-invasive virtual extractions of entire dentition (i.e. crown and roots) were realized for a more accurate analysis.

**Figure 1:**
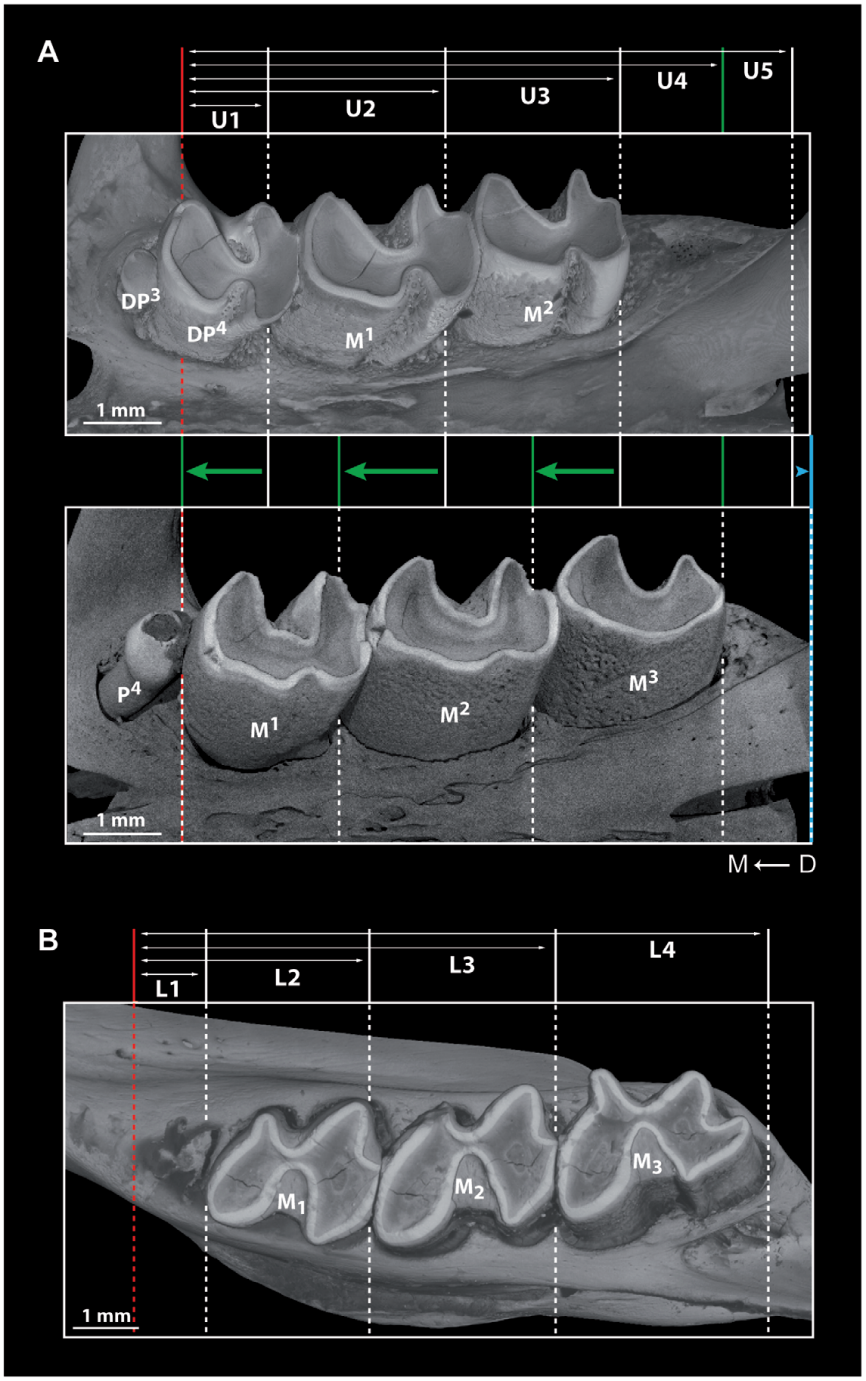
Characterization of dental mesial drift by using skull measurements (see material and methods for details). A, X-ray synchrotron and conventional microtomographic 3D rendering of left upper cheek tooth rows of *Pectinator spekei* (MNHN-CG1895-461 and 1995-19); green arrows indicate dental drift, blue arrow indicates maxillary growth. B, X-ray synchrotron microtomographic 3D rendering of left lower cheek tooth row of *Felovia vae* (MNHN-CG 1989-22). D → M stands for distal to mesial direction.

X^n^ and X_n_ respectively refer to the n^th^ upper and n^th^ lower cheek tooth, and Xn for both. Dental measurements were taken from the right upper dentition (U1–U5, Fig. 1) and right lower dentition (L1–L4, Fig. 1) to test the hypothesis of mesial drift. The mesial-most point of the lower dentition and the posterior part of the zygomatic arch for the upper dentition represent the starting points (i.e. references) and the mesial or distal base of molars the final points for each measurement, which were calculated with LAS Core (Leica®) software. Premolars were not included in such measurements because of their loss or replacement, contrary to molars which are not affected. Variations of U5 correspond to the maxillary growth. Skull lengths were also measured with a caliper to characterize the developmental stages. Variations of each distance were examined by linear regressions. Occurrence of drift was assessed by checking if the linear regression slopes are equal (null hypothesis: measurements are constant) or different from zero. Significant differences observed from Student’s t-test indicated that slopes could be used to characterize the presence and the relative importance of mesial drift.

## Results

### Overall Dental Characteristics of Extant Ctenodactylid Rodents

**Figure 2:**
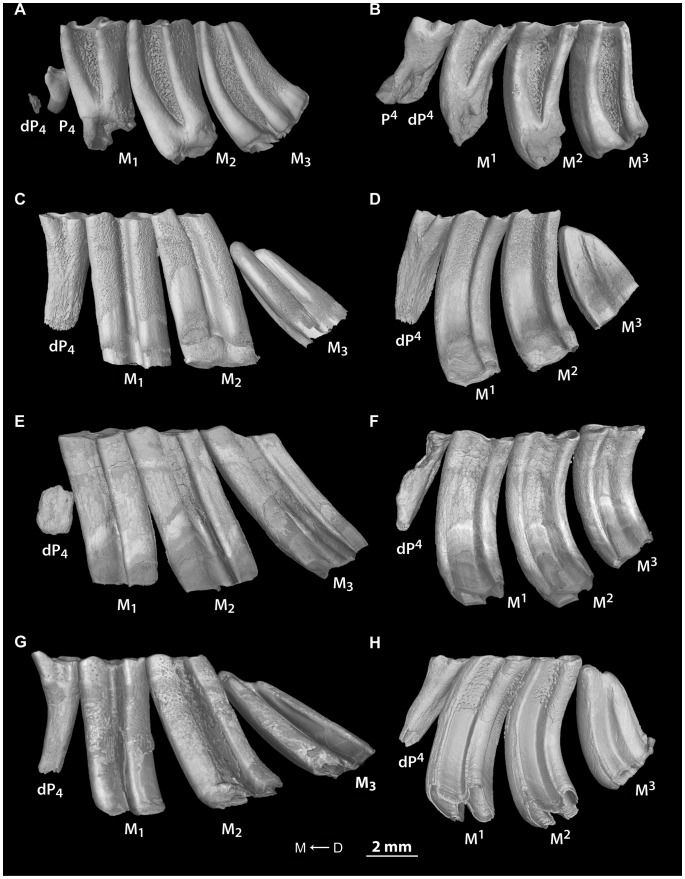
Lower and upper cheek tooth rows of each extant ctenodactylid in lateral view. X-ray synchrotron microtomographic 3D renderings of A–B, *Pectinator spekei* (MNHN-CG1893-226), C–D, *Ctenodactylus gundi* (MNHN-CG1986-255), E–F, *Felovia vae* (MNHN-CG1989-22) and G–H, *Massoutiera mzabi* (MNHN-CG1955-2). D → M stands for distal to mesial direction.

Here, we strictly focused our observations on the number and replacement of cheek teeth for each genus of extant ctenodactylids (Fig. 2). Dental occlusal morphologies have not been described here since they have already been accurately studied in previous works [18], [19], [21].

Dental formula of *Pectinator*: dP^3–4^/dP_4_, P^4^/P_4_, M^1–3^/M_1–3_ (Fig. 2A–B)

The investigated neonate skull (misidentified specimen: “*C. gundi*” MNHN-CG2006-198, (e.g. [18])) includes alveoli of dP^3^, in addition to dP4 and M1, but it does not display any evidence for the presence of dP_3_. One juvenile specimen (MNHN-CG 1895-461, Fig. 1A) indicates that dP^3^ is brachydont (i.e. low crowned) and single-rooted, while dP^4^ is three-rooted and includes a wide lingual root. Upper molars also have three roots which tend to merge and are highly reduced on M^1^ and M^2^ because of their strong hypsodonty (i.e. high crown). However, they are not euhypsodont (i.e. without root, [36], [37]), as roots are still present. With regard to the lower teeth, dP_4_ possesses one mesial and one distal root, and molars have two reduced roots, and the same morphological trends as seen on upper molars are observed. Slightly older specimens (MNHN-CG1981-503 and 1995-19) show that dP4 are replaced by smaller and single-rooted P4 after the full eruption of hypsodont M3, and that dP^3^ is shed. The tooth present in front of P_4_ is definitively not a P_3_, but it rather corresponds to the mesial fragment of dP_4_ which is not totally resorbed. The permanent premolar is indeed smaller that the deciduous one, and when erupting, it does not contribute to the complete shedding of the latter. Cementum is present on enamel of molars and dP4, notably in crown folds; a very thin layer partially surrounds the teeth. The dental formula of both adult and old specimens includes a P4, and three molars (M1-3) in each jaw quadrant.

Dental formula of *Ctenodactylus*: dP^4^/dP_4_, M^1–3^/M_1–3_ (Fig. 2C–D)

Lataste [27] was the first to pay attention to the sequence of dental eruption in *Ctenodactylus*. He established that the genus possesses dP4, P4, and three molars (M1-3). He also defined seven chronological stages concerning the sequence of dental eruption. The youngest specimen (1^st^ stage) presents erupting dP4 and M1. According to him, P^4^ is present from the 4^th^ stage, erupts after M^3^, and is lost at the 6^th^ stage. Our analysis of Lataste’s skulls permitted to establish that the “tiny tuberculous” P^4^ observed by Lataste is in fact a worn dP^4^ (e.g. MNHN-CG1963-921). Among the whole sample of investigated *Ctenodactylus gundi*, no specimen possesses either a P^4^, or a P_4_. Similarly, Vianey-Liaud et al. [18] did not find any specimen with P4. During the adulthood, the deciduous premolars are lost without being replaced; the older dentition thus comprises only molars in addition to the incisor. More generally, all the cheek teeth are euhypsodont, their seemingly single root remaining open during their whole life. They are homogeneously covered by a very thin layer of cementum.

Dental formula of *Felovia*: dP^4^/dP_4_, M^1-3^/M_1-3_ (Fig. 2E–F)

Several authors noted the presence of P^4^ and possibly P_4_ in this genus. The studied specimens indicate that only dP^4^ and dP_4_ are present. The presence of mesial alveoli is due to the loss of dP^4^, which occurs late as in *Ctenodactylus*. No skull of a neonate or of a young specimen could be studied. All the premolars are single-rooted, and molars are euhypsodont. Cementum strongly fills enamel crown folds, while the covering of the whole tooth is thinner and more heterogeneous.

Dental formula of *Massoutiera*: dP^4^/dP_4_, M^1–3^/M_1–3_ (Fig. 2G–H)

As for *Ctenodactylus* and *Felovia*, the presence of P^4^ and possibly P_4_ has been proposed for *Massoutiera*. The investigation of specimens indicated that, as for the two former genera, only dP^4^ and dP_4_ are present in addition to molars, and they are then lost as well. No dP^3^ was observed in the youngest specimen, which nonetheless possesses erupting dP4 and M1. All the premolars are single-rooted, and molars are euhypsodont. Although cementum is slightly thicker in enamel crown folds, the covering of tooth is relatively more homogeneous than in *Felovia*.

### Evidence of Mesial Drift in Ctenodactylidae

#### Measurements

**Figure 3:**
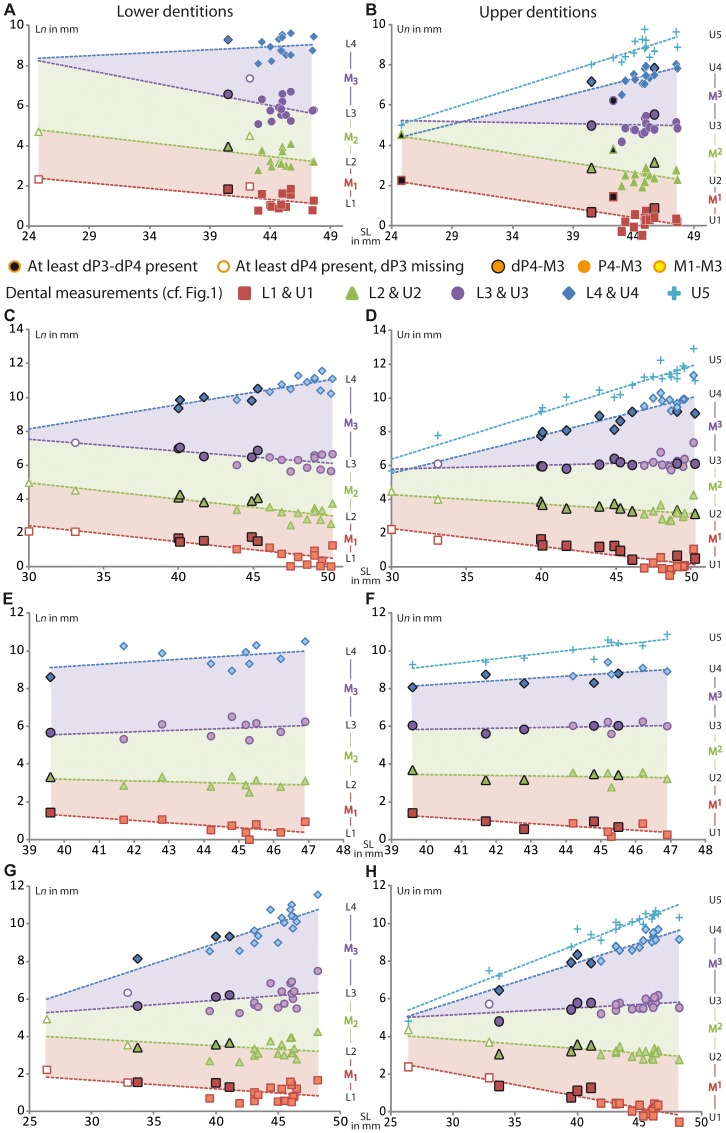
Graphs including skull measurements highlighting mesial drift for each extant ctenodactylid. A–B, *Pectinator spekei*, C–D, *Ctenodactylus gundi*, E–F, *Felovia vae* and G–H, *Massoutiera mzabi*. L*n*: measurements for right lower dentition; U*n*: measurements for right upper dentition, SL: Skull length. Red, green and violet areas show the evolution of M1, M2 and M3 length respectively, compared to skull length.

Measurements on lower and upper dental rows showed that mesial drift occurs in all species (Fig. 3, Table 1, Table S1). The displacement of teeth is obvious for first molars, since there is a significant diminution of measurements involving both mesial (L1, U1) and distal sides of M1 (L2, U2; Table 1), which is emphasized by negative values for upper dentitions (Fig. 3B, D, H), except for *Felovia* (Fig. 3F). Such observations are less marked in *Felovia* (L2, U2, Fig. 3E–F) and in lower molars of *Massoutiera* (L2, Fig. 3G), perhaps because we lack juvenile specimens for these genera. A drift of second molars occurs as well (L2, U2). However, the decrease of the measurements is only significant for the distal part of M_2_ (L3) of *Pectinator* and *Ctenodactylus* (Fig. 3A, C, Table 1). The other measurements of M2 (L3, U3) are nearly similar from juvenile to adult forms, since the slopes of linear regression are not significantly different from 0 in *Felovia* (Fig. 3E–F) and for M^2^ of *Pectinator* and *Ctenodactylus* (U3, Fig. 3B, D), while there is a slight significant increase of values in *Massoutiera* (Fig. 3G–H, Table 1). This result should be linked to the extended growth of teeth, which induces a slight enlargement of molars, and this might weaken the observable effect of mesial drift. The important growth of M3 can be noticed with a significant increase of measurements (L4, U4), and it is concomitant with both the maxillary growth (U5) and the dentary growth (proportional to L4).

**Table 1:** Data of linear regressions and Student’s t-test calculated for on each variable for each species.

		L1	L2	L3	L4	U1	U2	U3	U4	U5
*Pectinator*	a	−0.055	−0.069	−0.115	0.030	−0.091	−0.094	−0.012	0.152	0.190
	t	2.852	2.995	10.893	1.313	4.077	4.123	0.491	4.567	9.773
	df	15	15	15	15	15	15	15	15	15
	p	**0.010**	**0.007**	**<0.001**	0.204	**0.001**	**0.001**	0.629	**<0.001**	**<0.001**
	b	3.753	6.496	11.079	7.596	4.417	6.772	5.521	0.642	0.319
*Ctenodactylus*	a	−0.095	−0.096	−0.071	0.142	−0.105	−0.057	0.023	0.223	0.275
	t	5.114	5.775	5.701	7.357	6.199	4.168	1.663	6.528	14.291
	df	20	20	20	20	20	20	20	20	20
	p	**<0.001**	**<0.001**	**<0.001**	**<0.001**	**<0.001**	**<0.001**	0.112	**<0.001**	**<0.001**
	b	5.287	7.859	9.652	3.903	5.434	6.009	5.123	−1.123	−1.854
*Felovia*	a	−0.126	−0.042	0.067	0.121	−0.120	−0.022	0.030	0.120	0.212
	t	2.552	1.008	1.058	1.384	2.868	0.514	0.900	2.558	4.813
	df	8	8	8	8	8	8	8	8	8
	p	**0.034**	0.343	0.321	0.204	**0.021**	0.621	0.394	**0.034**	**0.001**
	b	6.318	4.886	2.912	4.294	6.008	4.309	4.621	3.381	0.681
*Massoutiera*	a	−0.046	−0.036	0.050	0.219	−0.123	−0.050	0.036	0.212	0.256
	t	2.594	1.671	2.223	6.958	11.343	4.924	3.478	5.909	18.479
	df	19	19	19	19	19	19	19	19	19
	p	**0.018**	0.111	**0.039**	**<0.001**	**<0.001**	**<0.001**	**0.003**	**<0.001**	**<0.001**
	b	3.049	4.949	3.924	0.217	5.752	5.351	4.081	−0.563	−1.356

L*n* refers to measurements on right lower dentition; U*n* refers to measurements on right upper dentition; a: slope of linear regression, df: degree of freedom (significance at α<0.05 is indicated in bold), b: intercept.

#### Histological results

**Figure 4:**
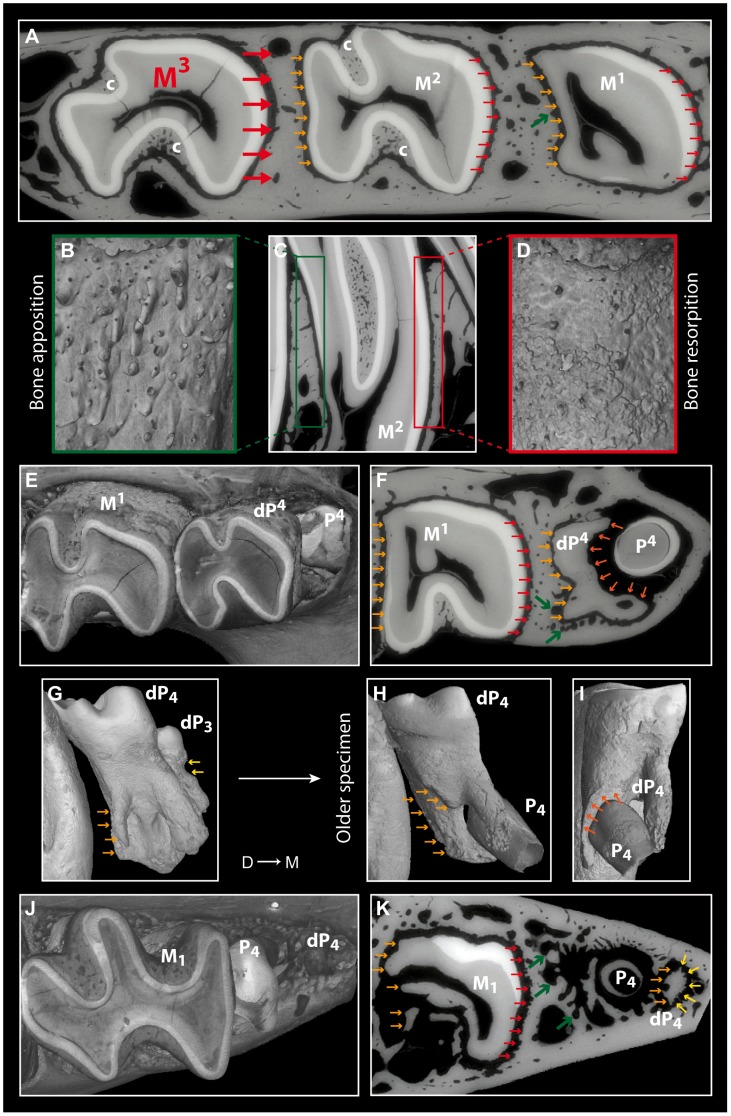
Bone and dental evidences of mesial drift in *Pectinator spekei*. A, F and K, X-ray synchrotron microtomographic virtual cross-section. C, X-ray synchrotron microtomographic virtual longitudinal section. B, D-E, G-J, X-ray synchrotron microtomographic 3D renderings. A-I, maxillary and upper cheek teeth (MNHN-CG1893-226 and 1895-461 for G); J-K, lower cheek teeth (MNHN-CG1893-226). Large red arrows display the orientation of compressive force; small red arrows indicate bone resorption by pointing the scalloped outline of alveolar wall; green arrows indicate bone apposition by pointing openings of vascular channels; small yellow to orange arrows indicate the different sites of dental resorption; c stands for cementum; D → M stands for distal to mesial direction.

Evidence of mesial drift can be observed in both bone and dental tissues (Fig. 4). *Pectinator* is the most striking case due to the loss of dP^3^ and the replacement of dP4 (Fig. 1). In virtual cross-section of upper cheek teeth (Fig. 4A), bone resorption is shown on mesial side of M^1^ and M^2^ by the serrated aspect of the distal part of interalveolar septa, and the entire alveolar wall shows an etched surface (Fig. 4C–D). Distally to teeth, bone apposition (or formation) is conversely illustrated by the presence of numerous openings of vascular channels in bone septa and alveolar surface (Fig. 4B–C). In this area, dental resorption occurs because the enamel layer is reduced to absent (see M^1^ and M^2^) and the outline is irregular due to the indirect compressive force of erupting distal molars. Dental resorption is strongly efficient at the mesial-most side of the cheek tooth row. Intense resorption notably affects deciduous premolars on various sides. In juveniles, mesial roots of dP^4^ tend to be resorbed by compressive forces induced by mesial drift (Fig. 4G). Resorption of dP^3^ is mesially obvious at the collar level, because of dental progression impeded by the physical constraints applied by the cortical bone delimiting the diastema, which is denser than the alveolar bone. In older specimens, dP^3^ is shed and P^4^ starts to protrude into the bone and leads to the mesial resorption of dP^4^ in addition to the mesial one resulting from mesial drift (Fig. 4E–F, H–I). Similar physiological mechanisms are observed on lower dentition (Fig. 4J–K). The remains of dP_4_, which stand in front of P4, are resorbed inside the bone by both distal and mesial compressions, and the remaining alveoli are filled with bone.

Similar impacts of mesial drift are noticed in *Ctenodactylus, Felovia* and *Massoutiera*. The pushing action of growing mesial molars is solely responsible for the loss of deciduous premolars resulting from an intense resorption (Fig. S1). In *Felovia* and *Massoutiera*, the layer of cementum is generally thicker on the mesial side than on the distal side of teeth, which is another consequence of dental migration.

## Discussion

### Importance of Premolar Development to Reassess Ctenodactyline Evolution

**Figure 5:**
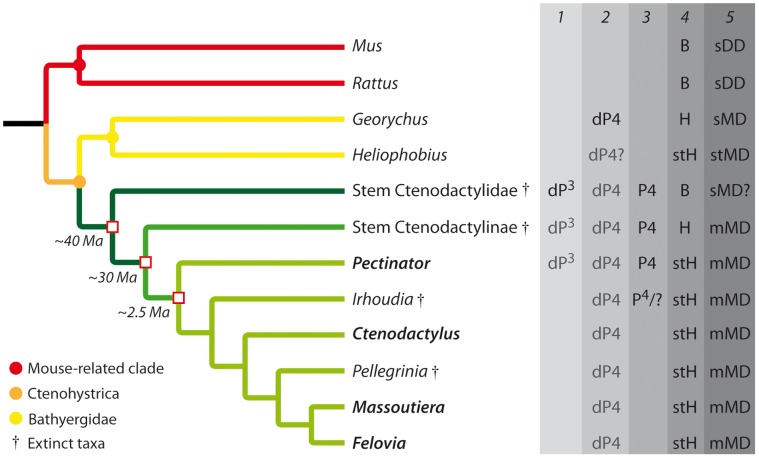
Simplified phylogeny of rodents showing main taxa bearing dental drift. Relationships between the main rodent clades were defined via a molecular analysis [61], while relationships within ctenodactylids were determined according to morphological analyses [18], [19]. 1, Presence of third upper premolar. 2, Presence of fourth deciduous premolars; 3, Presence of fourth definitive premolars. 4, Molar crown height: B Brachydont, H Hypsodont, stH strongly hypsodont. 5, Presence of dental drift: sDD slight Distal Drift, sMD slight Mesial Drift, mMD moderate Mesial Drift, stMD strong Mesial Drift. Green branches represent the Ctenodactylidae; in grey: tooth lost during lifetime.

We found that an important layer of cementum is present in crown folds of all extant ctenodactylines, including *Pectinator*, contrary to the results of previous studies [18], [19], [21]. Our study of extant ctenodactylids again demonstrated that *Pectinator* differs from other genera in being less hypsodont and in having a more primitive dental formula involving the presence of dP^3^, P^4^, and P_4_, which is consistent with its plesiomorphic dental morphology [18], [19], [21]. The tooth previously proposed as a dP_3_ actually corresponds to the anterior root of dP_4_, which remains in front of the erupted P_4_. Schrenk [38] studied the ontogenetic development of the skull of *Ctenodactylus gundi*. Interestingly, he noted the presence of a dP^3^ on several sections of the skulls, as in *Pectinator*. He indicated the presence of dP^3^ in embryos at stage 2 (Skull length: 12 mm) and stage 4 (Skull length: 22 mm). However, it does not seem that the incipiently mineralized tooth is indeed a dP^3^ at the stage 4, inasmuch as it is located behind the dP^4^. It might rather correspond to the mesial part of M^1^. The occurrence of a dP^3^ bud in *Ctenodactylus* means that this tooth starts to develop, but later regresses, contrary to *Pectinator*. Such abortion of tooth development prior to its mineralization has already been evidenced in the mouse and in a few squirrels [22], [23], [24]. This aborted tooth has also been hypothetically assigned to a premolar lost during evolution [26], [39]. Observation of unmineralized dP^3^ bud in these species is consistent with the fact this tooth was recently lost (i.e.; Pleistocene, 2.6 Ma-11,700 years) in *Ctenodactylus*, as in *Massoutiera* and *Felovia* [18] whose skull development has never been investigated. The developmental follow-up of such teeth is relevant for a better understanding of the evolutionary and developmental dynamics of the ctenodactyline dentition. In other words, it could inform about the real place of *Ctenodactylus* in the evolution of ctenodactylines, since it seems morphologically closer to the more primitive genus *Pectinator* [40], but it is phylogenetically closer to the more derived *Felovia-Massoutiera* clade ([19], Fig. 5).

Conversely to what has been proposed by numerous authors [19], [21], [27], P4 are absent in *Massoutiera*, *Felovia* and *Ctenodactylus*. It is worth mentioning that dP4 are more substantially worn in *Felovia*, *Massoutiera* and *Ctenodactylus* than they are in *Pectinator*, because of their retention in these genera. Thus, the teeth generally considered to be permanent premolars, due to their smaller size, actually correspond to worn deciduous premolars. As far as we know, one cannot say whether a beginning of development occurs for permanent premolars, which could be then stopped before mineralization.

### Interest of Dental Drift in Rodent Evolution with a Special Focus on the Ctenodactylid Radiation

Dental mesial drift occurs in all extant ctenodactylids. This mechanism involves premolars and the first two molars, while M3 likely represent the main forward pressures by both growing and erupting. The hypothesis of a pressure originating from distal molars can be supported by the fact that M2 could also exert pressure. For instance, the growth of these teeth leads to the dP^3^ loss in *Pectinator*, while M3 is just starting to develop. The mode of pressure could be notably illustrated by the mesial bending of M3 (Fig. 2), while erupting toward more mesial teeth. It can be considered that the bone locus dedicated to the dentition in ctenodactylids cannot include all the cheek teeth because of their greater length. Consequently, the eruption of distal molars can drive mesial drift because of their size. Similarly, Humans have an analogous problem because of their reduced jaw inducing a lack of places for wisdom teeth. More generally, we can assume that when mesial drift occurs in mammals, the length of the dentition (per jaw quadrant) is greater than the length of the corresponding bone locus due to either too large teeth (e.g. some primates and macropodids, and elephants) or extrateeth (e.g. manatees, silvery mole-rats, and pygmy rock wallabies). Such mechanism in ctenodactylids corresponds to a moderate mesial drift (mMD, Fig. 5) since only one tooth per jaw quadrant is lost. In a different way, the silvery mole-rat (*Heliophobius*) displays a strong mesial drift (stMD; Fig. 5), because many teeth are shed as the consequence of new teeth constantly erupting. At the opposite, the Cape mole-rat (*Georychus*) displays a slight mesial drift (sMD, Fig. 5) involving a weak displacement of teeth without dental loss [14].

To date, the above-mentioned species constitute the only known rodents having mesial drift. It is worth noticing that they all possess hypsodont teeth. This dental character probably favors the presence of mesial drift in rodents. Hypsodonty is the major evolutionary trend in rodents to withstand the effect of intensive wear. Therefore, mesial drift represent a superimposed mechanism partly originating from the significant enlargement of the occlusal surface of high-crowned molars during wear in ctenodactylids, whereas it is related to supernumerary teeth in the silvery mole-rat. It is also linked to the reduction of premolar size, affecting first the permanent ones, during the course of ctenodactylid evolution (i.e. since the Oligocene, [18]).

Such innovation also represents another means to withstand an important component of abrasive matter found in both plants and exogenous particles. Indeed, extant ctenodactylids generally usually live in rocky slopes and crevices in desert or semi-desert areas [41], where silica phytoliths present in grasses, and dust on the herbaceous layer are the most abundant sources of abrasive matter. This adaptation could appear early in the ctenodactylid evolution, since the Miocene and the radiation of first ctenodactylines [19], whose dentition is marked by the reduction of premolar size and the spread of semi-hypsodont forms (i.e equal height between lingual and vestibular sides, [18], [42], [43]). The first apparent evidence of mesial drift can be observed during the Upper Miocene, in *Metasayimys jebeli,* which shows strongly reduced P4 compared to dP4, and loss of dP^3^ during growth [21], as in *Pectinator*. This evolutionary trend could be parallel with the concomitant and massive expansion of grasses in open environments during the Miocene [44], [45].

According to some authors, a few rodents, such as laboratory rats and mice, display slight distal drift (sDD, Fig. 5) of molars [46], [47], [48]. It was suggested that this migration corresponds to the posterior lengthening of the jaw during development [49]. One can consider that without any assumption of effective pressure, this dental displacement is the result of a distal shift (i.e., virtual displacement) involving only bone growth [33], rather than a true drift. Nonetheless, bone remodeling of alveolar sockets was clearly demonstrated in such cases. It is difficult to assume that masticatory movements are mainly involved in dental migration because of their propalinal direction of mastication (from posterior to anterior side; [9]). Consequently, distal shift is probably the main component of displacement, which induces slight losses of approximal contact between molars leading to drift to recover contact, as demonstrated when a tooth is experimentally moved [46]. However, further studies are needed to understand the actual origin of physiological distal drift in these rodents, to be then compared to the characteristics of mesial drift from evolutionary and biological points of views.

### Concluding Remarks on Dental Drift Involving Biological and Biomedical Prospects

Rats and mice, in addition to rabbits, monkeys, cats and dogs, are frequently used for addressing orthodontics issues [48], [50], [51]. A closed coil spring is generally applied on animal’s dentition to study *in vivo* the impact of tensile forces. Such studies permit the evaluation of the overall consequences of orthodontic drift on the dentition, as the rates of bone remodeling and dental resorption for biomedical perspectives. Various orthodontic appliances are used in humans to withstand physiological mesial drift of teeth frequently leading to dental misalignments. In order to be as efficient as possible, their characteristics are previously defined by means of such experimental drift. More generally, the effects of orthodontic drift (artificial or experimental forces) are more accurately studied than those of physiological drift (natural forces) [52], while a better knowledge of this last component is useful for both biological and biomedical studies. In addition, orthodontic drift only involves tensile forces, whereas physiological drift is induced by compressive forces as observed in rodents. As a result, it is necessary to draw comparisons between these two mechanisms whose consequences could be slightly dissimilar at least at the histological level. In this way, a more precise investigation of mesial drift in rodents is needed.

The last noteworthy point concerns dental resorption resulting from the activity of odontoclasts, which is far less studied than bone remodeling driven by both osteoclasts and osteoblasts [53]. In most cases, the analysis of dental resorption refers to the eruption of permanent teeth replacing deciduous teeth by resorbing their roots [54], [55], but it can also be linked to orthodontic drift [56]. This last mechanism, when mesially oriented, frequently leads to mesial root resorption, whereas physiological mesial drift leads to distal root resorption. In addition to dentine and cementum, enamel can be affected by physiological mesial drift. Moreover, three different phases of dental resorption are noticed, when driven by compressive forces: (1) distal compression (mesial drift), (2) apical compression (dental eruption), and (3) mesial compression (the tooth reaches the end of the bone locus dedicated to the dentition, and it is an indirect consequence of mesial drift). The rodents showing mesial drift represent thus a rare opportunity to accurately study the resorption in the different point of tooth and on the various tissues constituting the tooth. That could also permit the proper assessment of the putative range of odontoclast activities which affect teeth, compared to osteoclast activities affecting bone [51], [57], [58]. Such prospects involving this new way of investigations in rodents could also be promising regarding other aspects of dental drift such as cementum repairs [59], the roles and status of periodontal ligament (i.e. responsible for teeth anchorage; [47], [60]) and transseptal fibers (i.e. linking adjacent teeth; [46]).

## Supporting Information

Figure S1 **Bone remodeling and dental resorption in upper dentition of Ctenodactylidae.** Synchrotron microtomographic virtual cross-sections of A, *Ctenodactylus gundi* (MNHN-CG1986-255), B, *Felovia vae* (MNHN-CG1989-22), and C, *Massoutiera mzabi* (MNHN-CG1960-3741). D → M stands for distal to mesial direction.
(TIF)

Table S1 **List of investigated specimens including data on dentitions for each Ctenodactylidae.** Abbreviations: L*n* refers to measurements on right lower dentition; U*n* refers to measurements on right upper dentition; MNHN: Museum National d’Histoire Naturelle; BSL: Basel (Naturhistorisches Museum). Symbols: [Xn] signifies the tooth is shed but the alveolus is still present; (Xn) means the tooth is erupting.
(XLS)
