# Supplementary figures and images for: Evolutionary and Biological Implications of Dental Mesial Drift in Rodents: The Case of the Ctenodactylidae (Rodentia, Mammalia)
Source: PLoS One. 2012 Nov 21;7(11):e50197. doi: 10.1371/journal.pone.0050197 (PMC3503976; doi:10.1371/journal.pone.0050197)

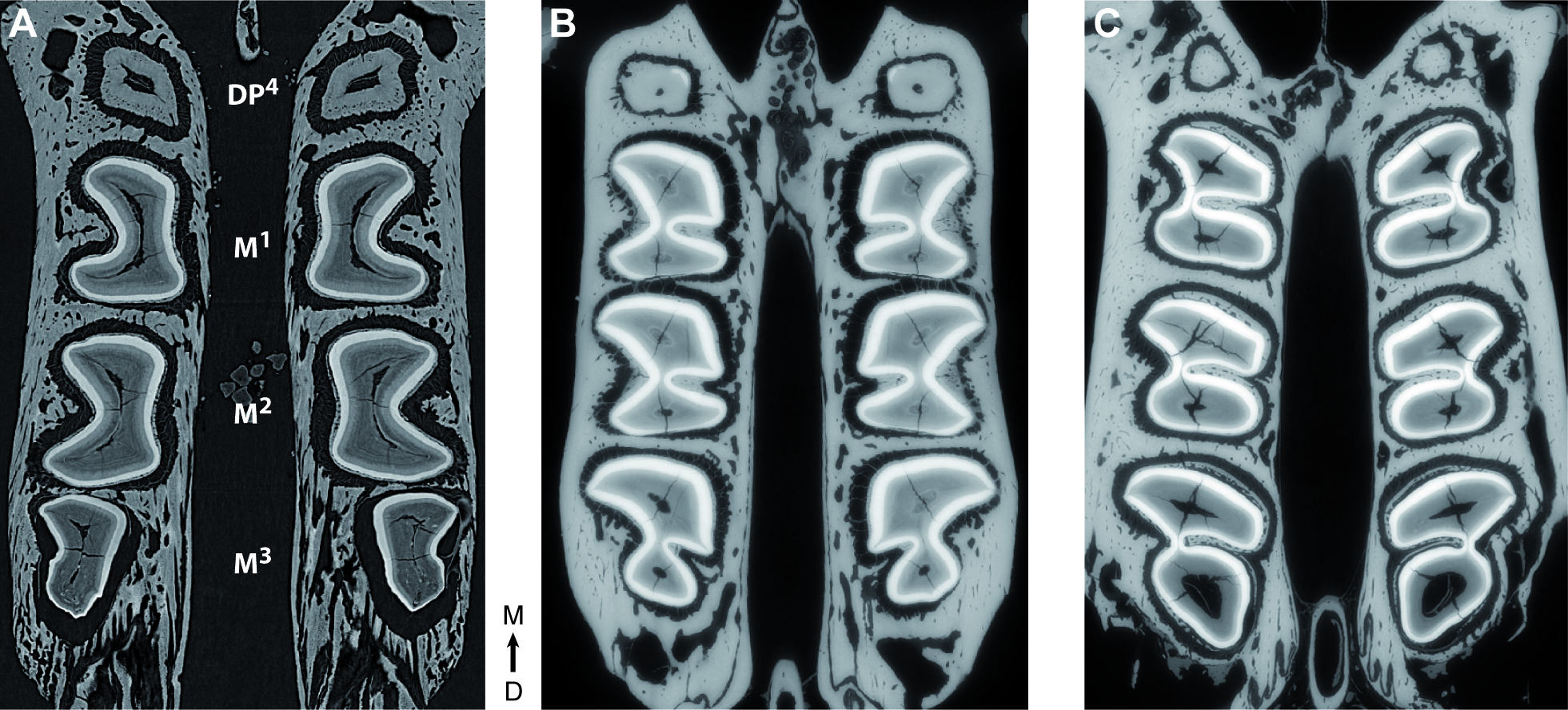

Supplement: Figure S1 — Bone remodeling and dental resorption in upper dentition of Ctenodactylidae. Synchrotron microtomographic virtual cross-sections of A, Ctenodactylus gundi (MNHN-CG1986-255), B, Felovia vae (MNHN-CG1989-22), and C, Massoutiera mzabi (MNHN-CG1960-3741). D → M stands for distal to mesial direction. (TIF) [file pone.0050197.s001.tif]
